# Viral surveillance of invasive mammals in New Zealand reveals unique viral lineages reflecting their introduction history

**DOI:** 10.1128/jvi.01440-25

**Published:** 2025-12-19

**Authors:** Rebecca K. French, Florian Pichlmueller, Stephanie J. Waller, Jeremy Dubrulle, Jess Tuxford, Andrew Veale, Jemma L. Geoghegan

**Affiliations:** 1 Department of Microbiology and Immunology, University of Otago 2495, Dunedin, New Zealand; 2 School of Biological Sciences, The University of Auckland 1415, Auckland, New Zealand; 3 University of Vienna, Centre for Microbiology and Environmental Systems Science 560173, Vienna, Austria; 4 Assure Quality, Mt Wellington, Auckland, New Zealand; 5 Manaaki Whenua Landcare Research, Lincoln, New Zealand; Cornell University Baker Institute for Animal Health, Ithaca, New York, USA

**Keywords:** metatranscriptomics, virome, RNA sequencing, *Wobbly possum disease virus*, *Ferret hepatitis E virus*, biosecurity

## Abstract

Introduced mammalian species in Aotearoa New Zealand pose significant ecological risks and may serve as reservoirs for novel or emerging infectious diseases. In this study, we present the first metatranscriptomic survey of viruses in five introduced mammals: ferrets (*Mustela furo*), stoats (*Mustela erminea*), weasels (*Mustela nivalis*), brushtail possums (*Trichosurus vulpecula*), and European hedgehogs (*Erinaceus europaeus*), sampled across both the North and South Islands. Through total RNA sequencing, we identified 11 mammalian-infecting viruses spanning eight viral families, including four novel virus species: *Ferret mastadenovirus, Possum astrovirus, Ferret pestivirus,* and *Weasel jeilongvirus*. Whole genomes were recovered for six of these viruses, enabling detailed phylogenetic analysis. Notably, we observed strong global geographic clustering in both *Wobbly possum disease virus* and *Ferret hepatitis E virus*, suggesting localized viral evolution following the introduction of their hosts into New Zealand. In addition, the detection of *Human rotavirus A* in hedgehogs highlights the possibility of reverse zoonotic transmission. Together, these findings broaden our understanding of the viral diversity harbored by New Zealand’s introduced mammals and provide a critical foundation for future biocontrol and disease surveillance efforts.

**IMPORTANCE:**

Introduced mammals in Aotearoa New Zealand not only threaten native biodiversity through predation and competition, but also represent a largely overlooked source of infectious disease risk. Viruses circulating in these species may spill over into native wildlife, livestock, or even humans, while human viruses can also establish in introduced animals and create new reservoirs. Understanding which viruses are present, and how they evolve in isolated host populations, is critical for anticipating future disease outbreaks, improving biosecurity, and guiding wildlife management strategies. This work provides foundational knowledge that links ecology, conservation, and health, highlighting the need to consider pathogens as part of the broader impact of invasive species.

## INTRODUCTION

Wild mammals are key reservoirs of zoonotic pathogens, posing significant risks to human health (1). These animals harbor a wide range of zoonotic and pathogenic viruses, including rabies virus (2), hantavirus (3, 4), morbillivirus (5), and coronaviruses (6). Due to their role in disease ecology, wild mammals have become a central focus of virological surveys, where hundreds of novel viruses have been uncovered across many hosts, including in bats, rodents, and shrews (7–9). These investigations have often identified viruses closely related to those that cause disease in humans or other animals (8), as well as uncovered novel viruses, many with zoonotic potential (1).

Largely isolated from the rest of the world for more than 50 million years, Aotearoa New Zealand has a unique ecosystem with only two species of bats as the only extant native terrestrial mammals (10). However, numerous mammalian species have been introduced into New Zealand, mainly by European settlers since the 19th century, including stoats, weasels, ferrets, hedgehogs, and possums (11). Invasions of mammalian species are one of the major causes of global biodiversity loss and ecosystem change (12), and in New Zealand, these mammalian pests have been responsible for both local and country-wide extinctions of many native animals due to predation (13). These mammals are also likely to harbor numerous viruses, either introduced via their native ranges or those that have emerged since their introduction. It is therefore likely that mammalian pests are reservoirs for a vast array of viruses, potentially posing a significant threat to endemic species with no prior exposure (14), as well as to public health. Biological control of pest animals, including the use of host-specific viruses, could be a key tool to eradicating mammalian pests in New Zealand (15, 16).

Very little is known about viruses in New Zealand mammals, with the primary focus for viral surveillance being limited to domestic animals. Viruses have been identified in New Zealand’s bats (17, 18), sea lions (19), long-finned pilot whales (*Globicephala melas*) (20), fur seals (*Arctocephalus forsteri*) (21), and Maui dolphins (*Cephalorhynchus hectori maui*) (22). Pathogenic viruses have also been identified in some wild introduced species, including *Rabbit hemorrhagic disease virus* in rabbits (23) and *Wobbly possum disease virus* in brushtail possums (24), as well as serological evidence that stoats are infected with multiple viruses (25). Nevertheless, the total assemblage of viruses (i.e., the virome) of many mammalian pests in New Zealand remains unexplored.

**Fig 1:**
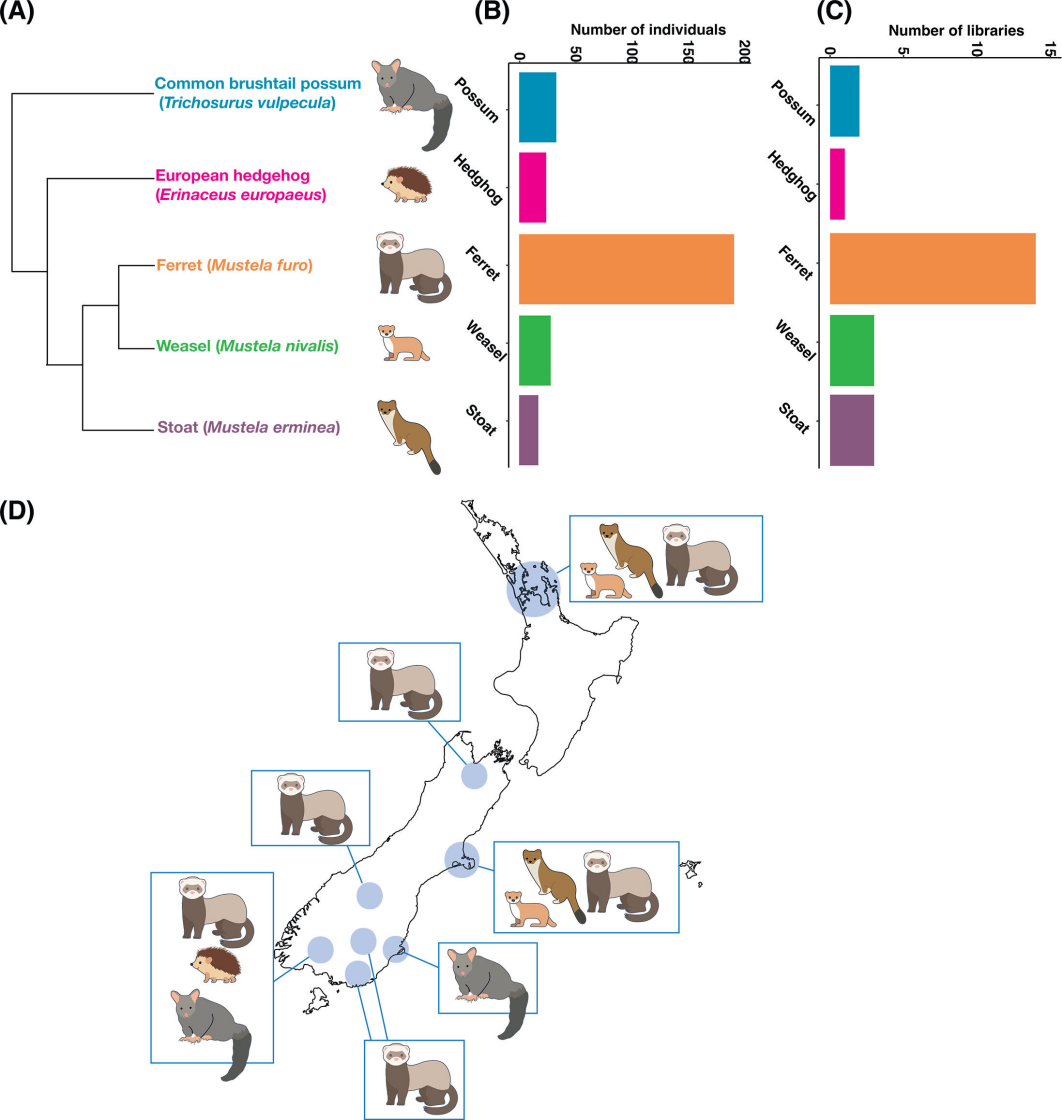
(**A**) Phylogeny of sampled mammals (based on the Open Tree of Life https://tree.opentreeoflife.org/). (**B**) The number of individual animals sampled per species. (**C**) The number of RNA-Seq libraries generated per species. (**D**) Map of New Zealand showing the sampling locations for each species.

Herein, we used total RNA sequencing to uncover the viruses harbored in five introduced mammals in New Zealand, including ferrets (*Mustela furo*), stoats (*Mustela erminea*), weasels (*Mustela nivalis*), brushtail possums (*Trichosurus vulpecula*), and European hedgehogs (*Erinaceus europaeus*), sampled across both the North and South Islands. Since ferrets, stoats, and weasels belong to the *Mustela* genus (Fig. 1A) and geographically overlap (26), we hypothesize that there is frequent cross-species virus transmission between these species compared to brushtail possums and hedgehogs. As the first virome-scale survey of introduced mammals in New Zealand, this study offers new insights into the evolutionary relationships and geographic distribution of mammalian viruses.

## RESULTS

**Fig 2:**
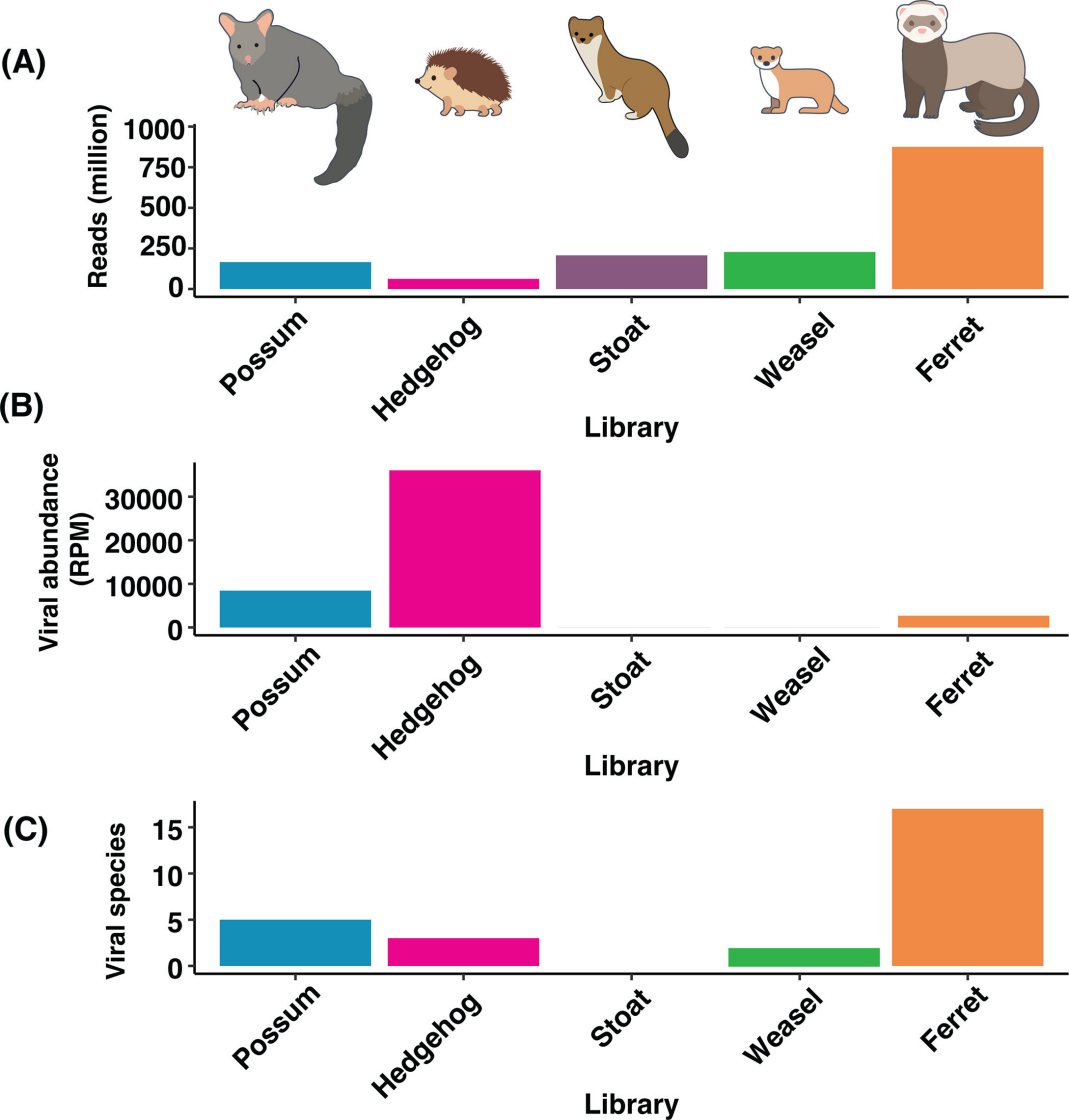
(**A**) The total number of reads (expressed in millions) for each host species sampled. (**B**) The total viral abundance (expressed in reads per million, RPM) per host species. (**C**) The number of viral species detected per host species.

We generated a total of 1.6 billion sequencing reads, with an average of 68 million per library (±11.2 million SD, Fig. 2). The hedgehog library had the highest viral abundance (>36,000 reads per million [RPM], Fig. 2; Table S2), while other libraries with high viral abundance included the two possum libraries (>800 RPM, Fig. 2; Table S2) and one ferret library (>1,500 RPM, Table S2). No viruses were detected in stoats sampled here.

We identified 20 virus transcripts that are known to infect mammals, spanning 11 virus species from eight viral families (Table 1), varying in abundance from 1 RPM (*Weasel jeilongvirus*) to >35,000 RPM (*Hedgehog hepatovirus*). Of these 11 virus species, four were considered novel since they shared <90% amino acid sequence similarity to other known viruses within the most conserved region (i.e., the polymerase). We recovered complete viral genomes from six virus species. We now describe the different groups of viruses in turn.

**TABLE 1:** Details of the viruses found in each RNA sequencing library^*a*^

Organism	Genbank accession	Host(s)	Library	Viral family	Genome type	Abundance	Novel	Gene(s)	Length
*Ferret mastadenovirus*	PX236813	Ferret	M1	*Adenoviridae*	ds DNA	4.8	Yes	DNA polymerase, partial CDS	936
*Hedgehog arterivirus*	PX236814	Hedgehog	M24	*Arteriviridae*	+ ss RNA	219	No	ORF1ab polyprotein, partial CDS	3,931
*Wobbly possum disease virus*	PX236815	Possum	M22	*Arteriviridae*	+ ss RNA	348	No	Whole genome	13,306
*Wobbly possum disease virus*	PX236816	Possum	M23	*Arteriviridae*	+ ss RNA	839	No	Whole genome	13,305
*Possum astrovirus*	PX236817	Possum	M22	*Astroviridae*	+ ss RNA	52	Yes	ORF1a Protein, ORF1b Protein, ORF2 Protein partial CDS	5,446
*Possum astrovirus*	PX236818	Possum	M23	*Astroviridae*	+ ss RNA	348	Yes	Whole genome	6,363
*Ferret pestivirus*	PX236819	Ferret	M15	*Flaviviridae*	+ ss RNA	81	Yes	Whole genome	11,472
*Ferret pestivirus*	PX236820	Ferret	M16	*Flaviviridae*	+ ss RNA	117	Yes	Whole genome	11,566
*Ferret pestivirus*	PX236821	Ferret	M17	*Flaviviridae*	+ ss RNA	38	Yes	Whole genome	11,468
*Ferret pestivirus*	PX236822	Ferret	M20	*Flaviviridae*	+ ss RNA	17	Yes	Whole genome	11,456
*Possum hepacivirus*	PX236823	Possum	M23	*Flaviviridae*	+ ss RNA	3382	No	Whole genome	9,122
*Ferret hepatitis E virus*	PX236824	Ferret	M14	*Hepeviridae*	+ ss RNA	391	No	Whole genome	8,733
*Ferret hepatitis E virus*	PX236825	Ferret	M15	*Hepeviridae*	+ ss RNA	360	No	Whole genome	8,737
*Ferret hepatitis E virus*	PX236826	Ferret	M16	*Hepeviridae*	+ ss RNA	119	No	Whole genome	8,740
*Ferret hepatitis E virus*	PX236827	Ferret	M17	*Hepeviridae*	+ ss RNA	1464	No	Whole genome	8,942
*Weasel jeilong virus*	PX236828	Weasel	M10	*Paramyxoviridae*	− ssRNA	1	Yes	RdRp, partial CDS	496
*Hedgehog hepatovirus*	PX236829	Hedgehog	M24	*Picornaviridae*	+ ss RNA	35,817	No	Whole genome	7,073
*Ferret parechovirus 2*	PX236830	Ferret	M5	*Picornaviridae*	+ ss RNA	1.44	No	Polyprotein, partial CDS	981
*Ferret parechovirus 2*	PX236830	Ferret	M16	*Picornaviridae*	+ ss RNA	0.15	No	Polyprotein, partial CDS	326
*Human rotavirus A*	PX236831	Hedgehog	M24	*Sedoreoviridae*	ds DNA	4.88	No	RdRp, partial CDS	379

^*a*^ Abundance is expressed in reads per million, and the length in nucleotides. CDS, coding sequence. RdRp, RNA-dependent RNA polymerase.

### Double-stranded DNA viruses

We identified two non-overlapping adenovirus fragments in ferrets sampled from the North Island, which we assume belong to the same virus (Fig. S1A). This adenovirus belongs to the *Mastadenovirus* genus, which we have provisionally named *Ferret mastadenovirus*. This virus was most closely related to *Polar bear adenovirus 1* (27), with 73% amino acid identity. In addition, a short fragment of *Human rotavirus A* from the *Sedoreoviridae* family was found in hedgehogs sampled from the South Island at low abundance (<5 RPM, Fig. S1D; Table 1). The virus could not be genotyped for G or P type due to the short fragment length (379 nucleotides); however, the closest known genetic relative was *Human rotavirus A* strain RVA/Human-tc/JPN/K8/1977/G1P[9] sampled in Japan, with 94% nucleotide sequence similarity. A small number of human transcripts were present in the hedgehog library (accounting for 1.33% of the total read count), thus it is possible that the detected rotavirus fragment originated from trace human contamination rather than active infection of the hedgehog. This level of human contamination was similar to all other libraries (which ranged from 0.75% to 5.33% human reads) where no human-infecting viruses were found.

### Single-stranded RNA viruses

**Fig 3:**
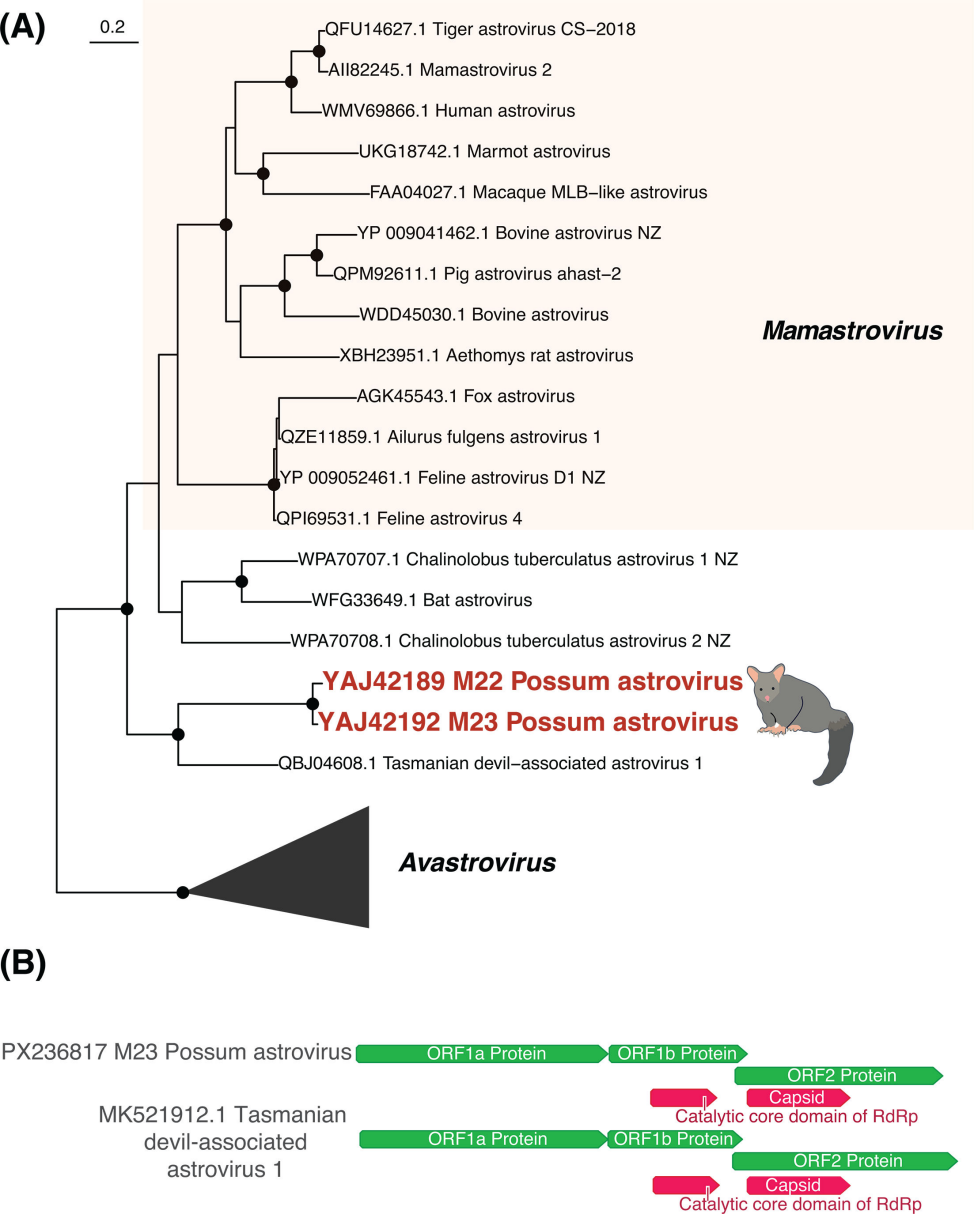
(**A**) Maximum likelihood phylogenetic tree of the *Astroviridae* (representative viruses only, *n* = 23, including collapsed clades) based on the ORF1b protein (alignment length of 343 amino acids post-trimming). Viruses identified in this study are shown in red and related viruses are shown in black. Black circles on nodes indicate bootstrap support values of >90%. Branches are scaled according to the number of amino acid substitutions per site, shown in the scale bar. The trees are midpoint rooted for purposes of clarity only. (**B**) Genome structure of *Possum astrovirus* and the closest known relative (*Tasmanian devil-associated astrovirus 1*). A more detailed annotated genome structure is displayed in Fig. S2A.

We identified an astrovirus in possums across two libraries sampled in the South Island, which we have provisionally named *Possum astrovirus*. A complete viral genome (6,363 nucleotides) as well as a near-complete genome (5,446 nucleotides) was obtained. These sequences were 86% similar at the nucleotide level, and the ORF1b proteins (containing the RNA-dependent RNA polymerase, RdRp) were 94% similar at the amino acid level. We therefore considered these sequences to be from the same virus species. Like other astroviruses, *Possum astrovirus* had an ORF1a (encoding the protease), ORF1b (RdRp), and ORF2 (capsid) (Fig. 3B; Fig. S2A). It also had the ribosomal frameshift (AAAAAAC) near the end of ORF1a, to create ORF1a/b. *Possum astrovirus* was most closely related to *Tasmanian devil*-*associated astrovirus 1* (28), with 56% amino acid sequence similarity. These two viruses formed a clade basal to the classified *Mamastrovirus* genus, but with strong bootstrap support (100%) (Fig. 3A).

**Fig 4:**
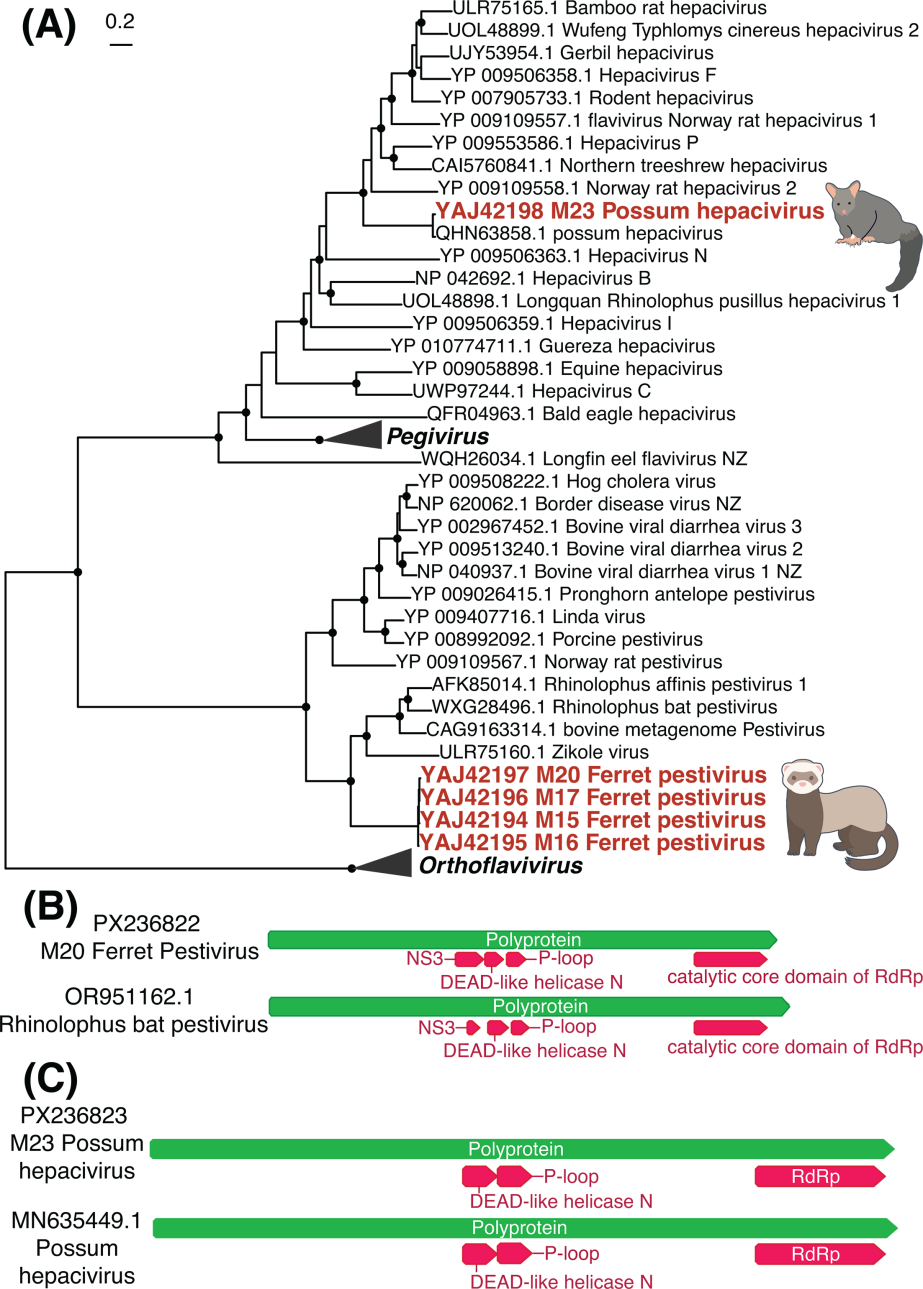
(**A**) Maximum likelihood phylogenetic tree of the *Flaviviridae* (representative viruses only, *n* = 47, including collapsed clades) based on the polyprotein (alignment length of 2,090 amino acids post-trimming). Viruses identified in this study are shown in red, while related viruses are shown in black. Black circles on nodes show bootstrap support values of >90%. Branches are scaled according to the number of amino acid substitutions per site, shown in the scale bar. The trees are midpoint rooted for clarity only. (**B**) Genome structure of *Ferret pestivirus* found in this study and a close relative (*Rhinolophus bat pestivirus*). A more detailed annotated genome structure is displayed in Fig. S2D. (**C**) Genome structure of *Possum hepacivirus* found in this study and *Possum hepacivirus* found previously in Australia. A more detailed annotated genome structure is displayed in Fig. S2B.

Five viruses across two viral species were found within the *Flaviviridae* in ferrets and possums. We identified four full genomes of a novel pestivirus in ferrets sampled from across the South Island, now termed *Ferret pestivirus*. These sequences were 93%–95% similar at the nucleotide level but were highly divergent from other pestiviruses with the closest known virus, *Rhinolophus affinis pestivirus 1*, sharing only between 42% and 47% amino acid sequence similarity (Fig. 4A). Like other pestiviruses, these genomes consisted of a single polyprotein approximately 11.4 kb in length (Fig. 4B). We also identified the whole genome of *Possum hepacivirus* at high abundance (>3,000 RPM, Table 1) with 96% amino acid and 85% nucleotide sequence similarity and the same genome structure (Fig. 4C) to the virus first identified in Australian brushtail possums (29).

**Fig 5:**
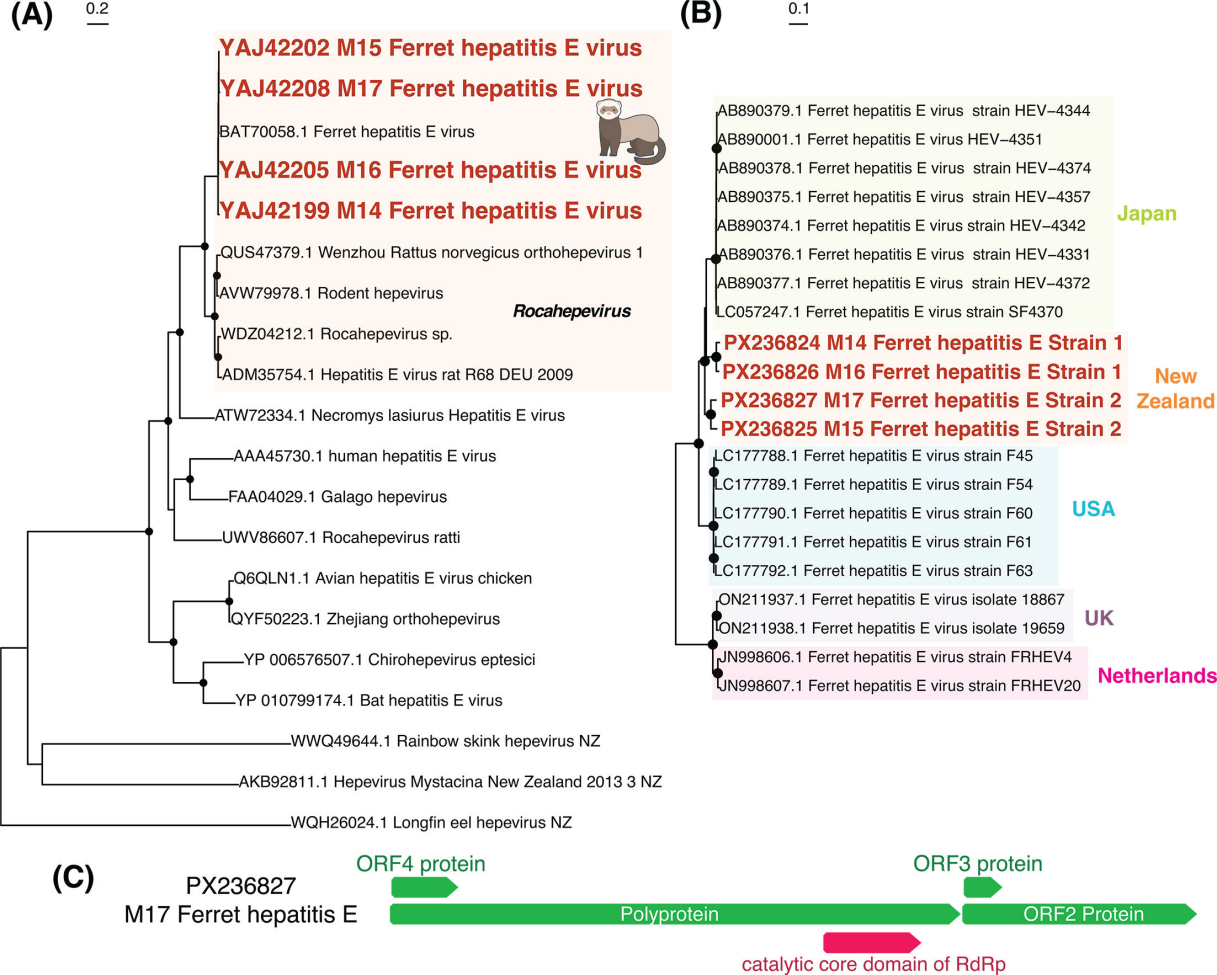
(**A**) Maximum likelihood phylogenetic tree of the *Hepeviridae* (representative viruses only, *n* = 20) based on the non-structural polyprotein (alignment length of 1,250 amino acids post-trimming). Viruses identified in this study are shown in red, and related viruses are shown in black. (**B**) Maximum likelihood phylogenetic tree of *Ferret hepatitis E* strains based on the whole genome at the nucleotide level. Shading denotes the country where the viruses were sampled. Black circles on nodes show bootstrap support values of >90%. Branches are scaled according to the number of amino acid (**A**) or nucleotide (**B**) substitutions per site, shown in the scale bar. The trees are midpoint rooted for purposes of clarity only. (**C**) Genome structure of Ferret hepatitis E virus was found in this study. A more detailed annotated genome structure is displayed in Fig. S2C.

Full genomes of *Ferret hepatitis E virus* were recovered from ferrets sampled from across the South Island, sharing between 92% and 98% amino acid sequence similarity with each other, and up to 92% similar to previously identified strains of this virus (Fig. 5). Phylogenetic analysis of the whole genome showed clear geographic clustering, with the New Zealand strains being most closely related to those sampled in Japan (30) (Fig. 5B). Within New Zealand, there appear to be two distinct strains (denoted strain 1 and 2), with 86%–88% nucleotide sequence similarity between strains, and 95%–96% nucleotide sequence similarity within the strains. Strain 1 was found in the lower and upper parts of the South Island (Southland and Marlborough), while strain 2 was found closer to the center of the South Island (Canterbury).

**Fig 6:**
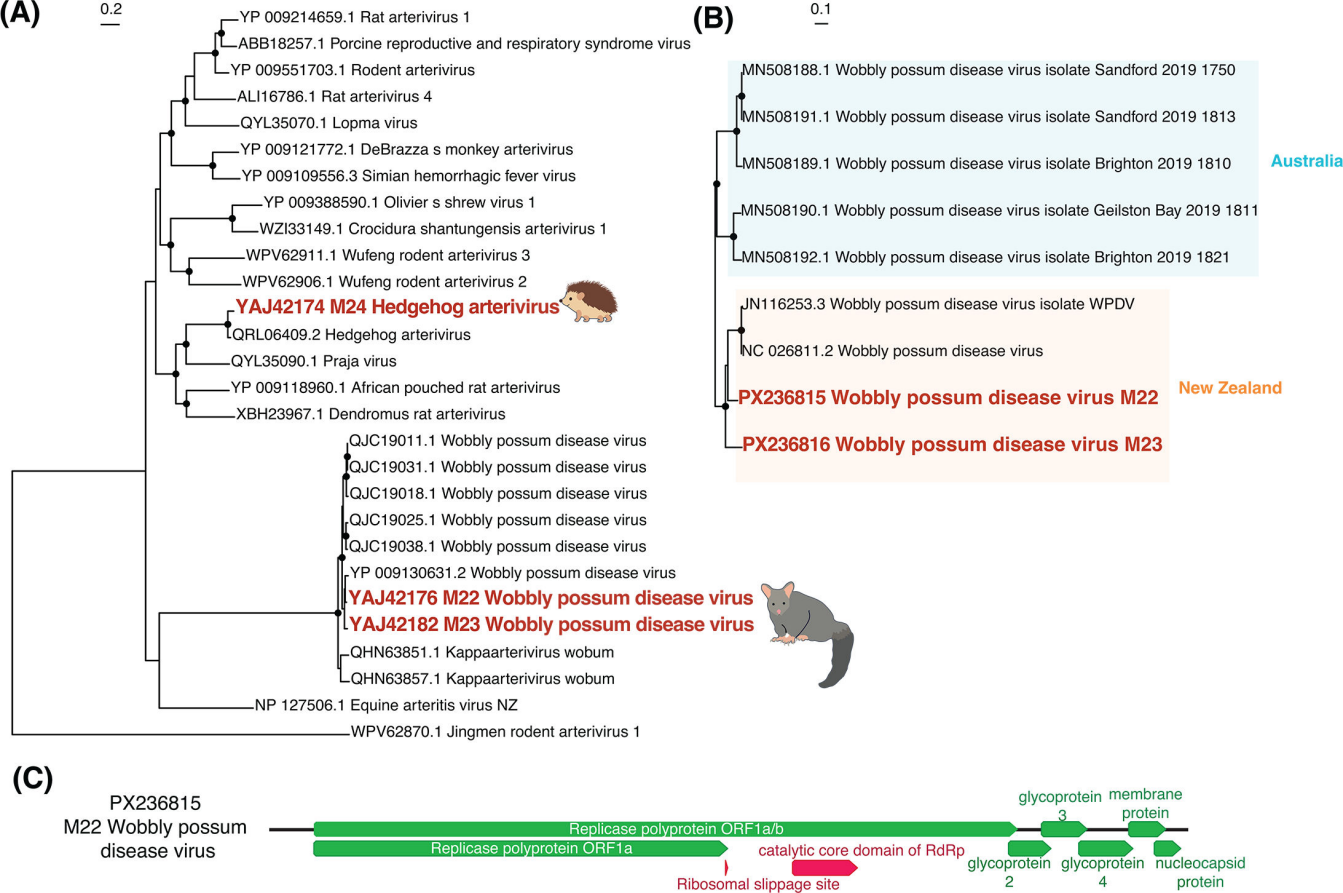
(**A**) Maximum likelihood phylogenetic tree of the *Arteriviridae* (representative viruses only, *n* = 28) based on the ORF1a/b polyprotein (alignment length of 1,194 amino acids post-trimming). Viruses identified in this study are shown in red, while related viruses are shown in black. (**B**) Maximum likelihood phylogenetic tree of WPDV strains based on the whole genome at the nucleotide level. Shading denotes the country in which the viruses were identified. Black circles on nodes show bootstrap support values of >90%. Branches are scaled according to the number of amino acid (**A**) or nucleotide (**B**) substitutions per site, shown in the scale bar. The trees are midpoint rooted for purposes of clarity only. (**C**) Genome structure of the WPDV found in this study. A more detailed annotated genome structure is displayed in Fig. S2E.

Within the *Arteriviridae*, we identified *Hedgehog arterivirus*, previously found in European hedgehogs in the United Kingdom (31), sharing 92% amino acid sequence similarity (Fig. 6A). We also found two whole genomes of *Wobbly possum disease virus* (WPDV) in possums sampled from the South Island, with 86% nucleotide sequence similarity to each other. These genomes had the same structure as other WPDV sequences, with a large ORF1a/b polyprotein and a frameshift at a ribosomal slippage site (Fig. 6C; Fig. S2E). Phylogenetic analysis of these viruses along with the seven already identified whole genomes showed that they clustered with WPDV previously identified in New Zealand in 1995, sharing 94%–96% amino acid with those previously identified (24) (Fig. 6B). The viruses we identified formed a clade with this New Zealand virus, while the WPDV found in Australia formed a sister clade (Fig. 6B).

Two virus species belonging to the *Picornaviridae* were identified in hedgehogs and ferrets. We found *Hedgehog hepatovirus* in very high abundance (>35,000 RPM, Table 1), with 98% amino acid sequence similarity to the virus previously found in European hedgehogs in Germany (32) (Fig. S1C). We also identified a novel parechovirus in two libraries of ferret samples, which we have provisionally named *Ferret Parechovirus 2*. The sequences in the two libraries do not overlap, yet we have conservatively assumed they are the same virus species (Fig. S1C). These viruses are most closely related to *Ferret parechovirus* found in ferrets in the Netherlands (33) with 78% and 88% amino acid sequence similarity, and *Parechovirus* sp. QAPp32 is found in the common pipistrelle bat, *Pipistrellus pipistrellus* (80% and 74% amino acid sequence similarity).

Finally, a virus from the genus *Jeilongvirus* in the *Paramyxoviridae* family was found in weasels sampled in the North Island and has been provisionally named *Weasel jeilongvirus*. We recovered three non-overlapping fragments of this virus, which we have conservatively assumed to be the same virus (Fig. S1B). These fragments are most closely related to *Feline paramyxovirus 163* found in Japan (84%–89%).

## DISCUSSION

This study presents the first metatranscriptomic survey of viruses in five introduced mammalian species in New Zealand: ferrets, stoats, weasels, brushtail possums, and hedgehogs. We identified 11 mammalian-infecting viral species spanning eight viral families, including four novel virus species within the *Adenoviridae, Astroviridae, Flaviviridae,* and *Paramyxoviridae*. Viral abundance varied considerably across species, with the highest viral load observed in hedgehogs (driven by *Hedgehog hepatovirus*), followed by possums and select samples of ferrets. Notably, complete genomes were recovered for six viruses, underscoring the utility of metatranscriptomics for in-depth viral discovery. These findings expand our understanding of the mammalian virome in New Zealand and highlight previously unrecognized viral diversity in introduced hosts.

### Novel viruses

With the increasing use of metatranscriptomics, novel viruses are frequently being discovered, including in mammalian hosts (9). Four novel viruses were identified in this study: *Ferret mastadenovirus*, *Possum astrovirus*, *Ferret pestivirus,* and *Weasel jeilongvirus*. These viruses were phylogenetically distinct from known genetic relatives and, in some cases, highly divergent, showing less than 60% amino acid sequence similarity, uncovering previously uncharacterized viral lineages. Genetic relatives of these viruses include known animal and zoonotic pathogens, such as Human astrovirus VA1/HMO-C associated with gastroenteritis, and closely related to bovine and mink astroviruses (34). While no clear zoonotic threats were identified in our data, these findings provide useful surveillance for public and animal health, particularly given the ecological overlap of these mammalian hosts and their proximity to humans, native wildlife, and domestic animals.

### Known viruses: historical introductions and long-term viral persistence

Several viruses identified in this study have been previously described, including WPDV, *Possum hepacivirus*, *Ferret hepatitis E virus,* and *Hedgehog arterivirus*, all sampled in animals from around the world (29, 35, 36). The detection of these viruses in New Zealand suggests long-term persistence following a single introduction along with their hosts in the 19th and 20th centuries. Population bottlenecks caused by small founding population sizes (37–39) could have eliminated many viruses from these mammal populations at the time of their introduction to New Zealand, but our results indicate that at least some viruses were introduced to New Zealand along with their hosts. Although these host populations are geographically isolated from their overseas counterparts, they often occur at high local densities within New Zealand (39), which would provide sufficient contact rates to maintain viral circulation over time. Notably, we also identified *Human rotavirus A* in a hedgehog sample, which raises the possibility of reverse zoonotic transmission, likely via environmental contamination. To our knowledge, *Human rotavirus A* has not been found in hedgehogs previously; however, hedgehogs are increasingly being recognized as potential reservoirs for zoonotic viruses (40), and their presence in urban or peri-urban environments could facilitate such spillovers. Human rotaviruses have occasionally been detected in non-human animals, including domestic animals and non-human primates (41, 42), lending weight to the possibility that reverse zoonotic transmission has also occurred in this case. However, it is also possible that the *Human rotavirus A* detected in the hedgehog library reflects contamination during sample collection or processing, rather than true infection. The nature of our sampling method (sourcing animals from established trapping programs) meant that human contamination was unavoidable. Further sampling of hedgehogs coupled with targeted PCR, *in situ* hybridization, or serology would be necessary to determine whether active infection occurred.

The phylogenetic patterns observed in both WPDV and *Ferret hepatitis E virus* revealed clear geographic clustering of New Zealand viruses. The WPDV genomes identified in this study clustered with the previously described New Zealand strain (24), forming a distinct clade sister to Australian WPDV lineages (29). This pattern is consistent with historical records of possum introductions, which occurred only in the early 1900s (11), and indicates that WPDV has likely been circulating and evolving within New Zealand possum populations since. Similarly, *Ferret hepatitis E virus* from New Zealand formed a well-supported clade most closely related to Japanese strains (30), suggesting a shared origin or common ancestral introduction. The distinct separation of these viruses by geographic region reflects host movement restrictions and ecological isolation, as well as evolutionary divergence following limited introduction events. Such patterns underscore the importance of localized viral surveillance and suggest that introduced mammalian species in New Zealand may harbor uniquely evolved viral lineages with potential implications for both animal health and biosecurity. These patterns also suggest that the strong biosecurity measures in New Zealand have successfully prevented further introductions of these mammals from overseas.

### Limited viral sharing among mustelid species

Despite the close evolutionary relationship and geographic overlap among mustelid species (26), we found no evidence of viral sharing between hosts. This result is surprising given the potential for cross-species viral transmission, particularly among mustelids (ferrets, stoats, and weasels). Evidence for spillover events of *Aleutian Mink Disease Virus* from mink (*Neogale vision*) was found in six other mustelid species in Poland (43) and eight in Finland (44), demonstrating the ability of some viruses to seemingly jump between mustelid species. The lack of evidence for cross-species viral transmission in our study may be an artifact of our opportunistic sampling, which included relatively few individuals from stoats and weasels compared to ferrets, reducing the probability of detecting low-frequency transmission events or shared virus species. Additionally, differences in habitat use (e.g., ecological niche separation [45]), behavior (solitary lifestyle [46]), or immune responses could contribute to barriers to viral exchange (47, 48). More extensive and systematic sampling across all species and regions is required to fully assess the potential for viral spillover between these hosts.

### Implications for biocontrol

New Zealand has the ambitious goal of becoming free of mammalian predators by 2050 (49). Biological control of pest animals, such as those sampled in this study, could be a key tool for reaching this goal (15, 16). Indeed, the discovery of host-specific viruses highlights intriguing possibilities for biological control. Nevertheless, for effective biocontrol, viruses need to be highly pathogenic, species-specific, and safe for non-target species—including humans, native wildlife, and domestic animals. All of the viruses we detected, including the novel pestivirus in ferrets and astrovirus in possums, were identified in apparently healthy individuals where no overt signs of disease were noted. The exception may be WPDV, which has been associated with neurological disease in brushtail possums (29), as well as *Hedgehog arterivirus*, associated with neurological disease and fatal encephalitis (35, 50), although as above, no animals sampled had obvious signs of disease. Rigorous assessment of pathogenicity and species specificity is required prior to any future application. More broadly, the use of viruses as biocontrol agents remains contentious (51) and would require robust risk assessment and regulatory oversight, especially in a biodiverse and conservation-sensitive context like New Zealand.

Our findings highlight several promising avenues for future research. First, increasing the sample size and geographic coverage for underrepresented species, such as stoats, weasels, and hedgehogs, would allow for a more robust assessment of viral diversity and potential cross-species viral transmission. Second, functional studies of the novel viruses identified here could help elucidate their host range, transmission potential, and pathogenicity. Third, integrating ecological data, such as species movement, diet, and habitat overlap, could shed light on the mechanisms shaping viral communities. Finally, monitoring for known and novel viruses in native New Zealand wildlife is essential to assess the potential for spillover from introduced species, particularly as efforts to manage or eradicate invasive mammals continue. Together, these findings underscore the value of viral surveillance in introduced wildlife and provide a crucial foundation for understanding and mitigating emerging infectious disease risks in New Zealand’s unique ecosystem.

## MATERIALS AND METHODS

### Sampling and RNA extraction

Kidney and liver samples were collected from 290 individuals from five introduced mammalian species: ferrets (*Mustela furo*), stoats (*Mustela erminea*), weasels (*Mustela nivalis*), brushtail possums (*Trichosurus vulpecula*), and European hedgehogs (*Erinaceus europaeus*), from the upper North Island and South Island of New Zealand in 2021 (Fig. 1). Animals were either live trapped and culled, kill trapped, or opportunistically collected as fresh roadkill. All animals that were trapped and killed were done so as part of already established pest control efforts. Upon collection, animals were frozen at −20°C and transferred to the University of Auckland (for North Island samples) or the University of Otago (for South Island samples). Before dissection, carcasses were thawed and checked for signs of decomposition to determine suitability for RNA extraction. Liver and kidney tissue samples were harvested and stored in RNALater (Thermo Fisher Scientific) at −80°C until RNA extraction using the Qiagen RNeasy Plus Mini kit (Qiagen) following the manufacturer’s instructions. RNA from up to 30 individuals from each species was pooled per location and ribosomal RNA was removed using the Ribo-Zero-Gold Kit from Illumina, then sequenced using Illumina NovaSeq 6000 at the Australian Genome Research Facility (AGRF), Melbourne, Australia.

### Viral discovery

Sequencing reads were quality-trimmed using Trimmomatic (v0.38), with removal of adapter sequences and bases with a quality score below 5 using a sliding window of four bases (52). Additionally, low-quality bases (quality score <3) were trimmed from the ends of reads, and sequences shorter than 25 nucleotides were discarded. Following quality control, reads were *de novo* assembled into contigs using MEGAHIT (v1.2.9) (53). These contigs were then compared to the NCBI nucleotide (nt) and non-redundant protein (nr) databases using BLASTn (BLAST+ v2.13.0 [54]) and DIAMOND (DIAMOND v2.1.6 [55]) to identify viral sequences. To reduce false positives, similarity thresholds of 1 × 10^−5^ for the nt database and 1 × 10^−10^ for the nr database were applied. Virus abundance was estimated by mapping reads back to the assembled contigs using Bowtie2 (v2.4.5 [56]) and SAMtools (v1.9 [57]). Viral sequences present at read counts <0.1% of those in another library and >99% identical at the nucleotide level were considered likely cross-contamination due to index hopping and excluded from further analysis. The probable host origin of each virus was inferred based on phylogenetic relatedness to known viruses. Viruses that clustered with known mammalian viruses were subject to further evolutionary analysis. Viruses that were phylogenetically distinct from vertebrate host viruses were assumed to be more likely associated with diet, microbiome, or environmental sources.

### Phylogenetic analysis

Putative viral transcripts were first translated and then aligned with representative protein viral sequences from the same viral genus or family retrieved from GenBank. These representative sequences were chosen to capture diversity within each viral genus or family by selecting a small number of sequences from major clades and including all sequences closely related to the viruses identified in this study. Alignments were performed using MAFFT (v7.402) (58) with the E-INS-i or L-INS-i algorithms (Table S1) and subsequently trimmed using TrimAl (v1.4.1) (59). Phylogenetic trees for each viral family or genus were estimated using the maximum likelihood method in IQ-TREE (v1.6.12), with the program determining the best-fit substitution model using ModelFinder (60) and node robustness evaluated through the approximate likelihood ratio test with 1,000 replicates (61). Sequences found within the same library were considered to represent different viral species (rather than intra-species viral diversity) if overlapping aligned sequences had <90% amino acid identity. Phylogenetic trees were visualized using APE (v5.4) (62) and ggtree (v2.4.1) (63) in R (v4.0.5) (64).

Selected whole viral genomes were also further analyzed at the nucleotide level. Annotations were conducted using BLAST and the conserved domains database (65). Phylogenetic analysis was conducted at the nucleotide level using whole-genome sequences, aligning the sequences using MAFFT. Phylogenetic trees were estimated using the same methods as described above.

## Data Availability

The sequence data generated in this study have been deposited in the Sequence Read Archive (SRA) under the project accession number PRJNA1304147. Virus consensus sequences have been deposited in NCBI/GenBank with assigned accession numbers PX236813–PX236831.

## SUPPLEMENTAL MATERIAL

Fig. S1 jvi.01440-25-s0001.pdf Maximum likelihood phylogenetic tree of the Adenoviridae, Paramyxoviridae, Picornaviridae, and Rotavirus.

Fig. S2 jvi.01440-25-s0002.pdf Annotated genome structures.

Table S1 jvi.01440-25-s0003.xlsx Alignment information for each viral family/group.

Table S2 jvi.01440-25-s0004.xlsx Library metadata.

ASM does not own the copyrights to Supplemental Material that may be linked to, or accessed through, an article. The authors have granted ASM a non-exclusive, world-wide license to publish the Supplemental Material files. Please contact the corresponding author directly for reuse.

## References

[1] Olival KJ, Hosseini PR, Zambrana-Torrelio C, Ross N, Bogich TL, Daszak P. 2017. Host and viral traits predict zoonotic spillover from mammals. Nature 546:646–650. doi:10.1038/nature22975 28636590 PMC5570460

[2] Akdesir E, Origgi FC, Wimmershoff J, Frey J, Frey CF, Ryser-Degiorgis M-P. 2018. Causes of mortality and morbidity in free-ranging mustelids in Switzerland: necropsy data from over 50 years of general health surveillance. BMC Vet Res 14:195. doi:10.1186/s12917-018-1494-0 29921290 PMC6009050

[3] Milholland MT, Castro-Arellano I, Suzán G, Garcia-Peña GE, Lee TE, Rohde RE, Alonso Aguirre A, Mills JN. 2018. Global diversity and distribution of hantaviruses and their hosts. Ecohealth 15:163–208. doi:10.1007/s10393-017-1305-2 29713899

[4] Dubrulle J, Kauffman K, Soarimalala V, Randriamoria T, Goodman SM, Herrera J, Nunn C, Tortosa P. 2025. Effect of land-use on hantavirus infection among introduced and endemic small mammals of madagascar. Ecol Evol 15:e70914. doi:10.1002/ece3.70914 40196405 PMC11975053

[5] Takeda M, Seki F, Yamamoto Y, Nao N, Tokiwa H. 2020. Animal morbilliviruses and their cross-species transmission potential. Curr Opin Virol 41:38–45. doi:10.1016/j.coviro.2020.03.005 32344228

[6] Qiu X, Liu Y, Sha A. 2023. SARS-CoV-2 and natural infection in animals. J Med Virol 95:e28147. doi:10.1002/jmv.28147 36121159 PMC9538246

[7] Chen Y-M, Hu S-J, Lin X-D, Tian J-H, Lv J-X, Wang M-R, Luo X-Q, Pei Y-Y, Hu R-X, Song Z-G, Holmes EC, Zhang Y-Z. 2023. Host traits shape virome composition and virus transmission in wild small mammals. Cell 186:4662–4675. doi:10.1016/j.cell.2023.08.029 37734372

[8] Wu Z, Lu L, Du J, Yang L, Ren X, Liu B, Jiang J, Yang J, Dong J, Sun L, Zhu Y, Li Y, Zheng D, Zhang C, Su H, Zheng Y, Zhou H, Zhu G, Li H, Chmura A, Yang F, Daszak P, Wang J, Liu Q, Jin Q. 2018. Comparative analysis of rodent and small mammal viromes to better understand the wildlife origin of emerging infectious diseases. Microbiome 6:178. doi:10.1186/s40168-018-0554-9 30285857 PMC6171170

[9] Xin G, Wang D, Zhang X, Cen Q, Peng M, Liao Y, Wang J, Le S, Cheng J, Wu W, et al.. 2025. Infectome analysis of small mammals in Southern China reveals ecological associations and emerging threats from diverse pathogens. bioRxiv. doi:10.1101/2025.02.09.637073 PMC12722744 41387678

[10] Wallis GP, Trewick SA. 2009. New Zealand phylogeography: evolution on a small continent. Mol Ecol 18:3548–3580. doi:10.1111/j.1365-294X.2009.04294.x 19674312

[11] King C, Forsyth D. 2021. The handbook of New Zealand mammals. Csiro Publishing.

[12] Courchamp F, Chapuis J-L, Pascal M. 2003. Mammal invaders on islands: impact, control and control impact. Biol Rev Camb Philos Soc 78:347–383. doi:10.1017/s1464793102006061 14558589

[13] Innes J, Kelly D, Overton JM, Gillies C. 2010. Predation and other factors currently limiting New Zealand forest birds. N Z J Ecol 34:86.

[14] Hale KA, Briskie JV. 2007. Decreased immunocompetence in a severely bottlenecked population of an endemic New Zealand bird. Anim Conserv 10:2–10. doi:10.1111/j.1469-1795.2006.00059.x

[15] Eason CT, Shapiro L, Ogilvie S, King C, Clout M. 2017. Trends in the development of mammalian pest control technology in New Zealand. N Z J Zool 44:267–304. doi:10.1080/03014223.2017.1337645

[16] McDonald RA, Lariviere S. 2001. Diseases and pathogens of Mustela spp, with special reference to the biological control of introduced stoat Mustela erminea populations in New Zealand. J R Soc N Z 31:721–744. doi:10.1080/03014223.2001.9517671

[17] Wang J, Moore NE, Murray ZL, McInnes K, White DJ, Tompkins DM, Hall RJ. 2015. Discovery of novel virus sequences in an isolated and threatened bat species, the New Zealand lesser short-tailed bat (Mystacina tuberculata). J Gen Virol 96:2442–2452. doi:10.1099/vir.0.000158 25900137 PMC4681071

[18] Waller SJ, Tortosa P, Thurley T, O’Donnell CFJ, Jackson R, Dennis G, Grimwood RM, Holmes EC, McInnes K, Geoghegan JL. 2024. Virome analysis of New Zealand’s bats reveals cross-species viral transmission among the Coronaviridae. Virus Evol 10:veae008. doi:10.1093/ve/veae008 38379777 PMC10878368

[19] Holdsworth NS, Weinzweig N, Lalas C, Waller SJ, Geoghegan JL, French RK. 2025. Uncovering the faecal virome of New Zealand sea lions (Phocarctos hookeri) reveals virus sharing among geographically distinct pinnipeds. Virology (Auckl) 610:110617. doi:10.1016/j.virol.2025.110617 40596795

[20] Van Bressem M-F, Waerebeek KV, Jepson PD, Raga JA, Duignan PJ, Nielsen O, Di Beneditto AP, Siciliano S, Ramos R, Kant W, Peddemors V, Kinoshita R, Ross PS, López-Fernandez A, Evans K, Crespo E, Barrett T. 2001. An insight into the epidemiology of dolphin morbillivirus worldwide. Vet Microbiol 81:287–304. doi:10.1016/S0378-1135(01)00368-6 11390111

[21] Wilson A, Gias E, Little A, Jauregui R, Low YS, Pulford D, Steyn A, Sylvester K, Green D, O’Keefe J, McCulley M. 2025. Genome sequence of a divergent strain of canine distemper virus detected in New Zealand fur seals. Microbiol Resour Announc 14:e00151–25. doi:10.1128/mra.00151-25 40293266 PMC12160475

[22] Van Bressem M, Van Waerebeek K, Aznar F, Raga J, Jepson P, Duignan P, Deaville R, Flach L, Viddi F, Baker J, Di Beneditto A, Echegaray M, Genov T, Reyes J, Felix F, Gaspar R, Ramos R, Peddemors V, Sanino G, Siebert U. 2009. Epidemiological pattern of tattoo skin disease: a potential general health indicator for cetaceans. Dis Aquat Org 85:225–237. doi:10.3354/dao02080 19750811

[23] Parkes JP, Norbury GL, Heyward RP, Sullivan G. 2002. Epidemiology of rabbit haemorrhagic disease (RHD) in the South Island, New Zealand, 1997–2001. Wildlife Research 29:543–555. doi:10.1071/WR00108

[24] Dunowska M, Biggs PJ, Zheng T, Perrott MR. 2012. Identification of a novel nidovirus associated with a neurological disease of the Australian brushtail possum (Trichosurus vulpecula). Vet Microbiol 156:418–424. doi:10.1016/j.vetmic.2011.11.013 22153843 PMC7117198

[25] McDonald RA, Birtles RJ, McCracken C, Day MJ. 2008. Histological and serological evidence of disease among invasive, non-native stoats Mustela erminea. Vet J 175:403–408. doi:10.1016/j.tvjl.2007.01.028 17434325 PMC7110800

[26] O’Donnell C, Weston K, Monks J. 2017. Impacts of introduced mammalian predators on New Zealand’s alpine fauna. NZ J Ecol 41:1–22. doi:10.20417/nzjecol.41.18

[27] Böszörményi KP, Podgorski II, Vidovszky MZ, Sós E, Benkő M, Harrach B. 2020. Full genome sequence analysis of a novel adenovirus from a captive polar bear (Ursus maritimus). Virus Res 277:197846. doi:10.1016/j.virusres.2019.197846 31870796

[28] Chong R, Shi M, Grueber CE, Holmes EC, Hogg CJ, Belov K, Barrs VR. 2019. Fecal viral diversity of captive and wild tasmanian devils characterized using virion-enriched metagenomics and metatranscriptomics. J Virol 93:00205–00219. doi:10.1128/JVI.00205-19 PMC6532096 30867308

[29] Chang W-S, Eden J-S, Hartley WJ, Shi M, Rose K, Holmes EC. 2019. Metagenomic discovery and co-infection of diverse wobbly possum disease viruses and a novel hepacivirus in Australian brushtail possums. One Health Outlook 1:5. doi:10.1186/s42522-019-0006-x 33829126 PMC7990097

[30] Li T-C, Yoshizaki S, Yang T, Kataoka M, Nakamura T, Ami Y, Yuriko S, Takeda N, Wakita T. 2016. Production of infectious ferret hepatitis E virus in a human hepatocarcinoma cell line PLC/PRF/5. Virus Res 213:283–288. doi:10.1016/j.virusres.2015.11.027 26763355

[31] Dastjerdi A, Inglese N, Partridge T, Karuna S, Everest DJ, Frossard J-P, Dagleish MP, Stidworthy MF. 2021. Novel arterivirus associated with outbreak of fatal encephalitis in European Hedgehogs, England, 2019. Emerg Infect Dis 27:578–581. doi:10.3201/eid2702.201962 33496231 PMC7853545

[32] Drexler JF, Corman VM, Lukashev AN, van den Brand JMA, Gmyl AP, Brünink S, Rasche A, Seggewiβ N, Feng H, Leijten LM, et al.. 2015. Evolutionary origins of hepatitis A virus in small mammals. Proc Natl Acad Sci USA 112:15190–15195. doi:10.1073/pnas.1516992112 26575627 PMC4679062

[33] Smits SL, Raj VS, Oduber MD, Schapendonk CME, Bodewes R, Provacia L, Stittelaar KJ, Osterhaus ADME, Haagmans BL. 2013. Metagenomic analysis of the ferret fecal viral flora. PLoS One 8:e71595. doi:10.1371/journal.pone.0071595 23977082 PMC3748082

[34] Cordey S, Vu D-L, Schibler M, L’Huillier AG, Brito F, Docquier M, Posfay-Barbe KM, Petty TJ, Turin L, Zdobnov EM. 2016. Astrovirus MLB2, a new gastroenteric virus associated with meningitis and disseminated infection. Emerging Infect Dis 22:846. doi:10.3201/eid2205.151807 PMC4861523 27088842

[35] Lean FZX, Stidworthy MF, Dastjerdi A, Partridge T, Smith S, Gough J, Núñez A, Lawson B, Seilern-Macpherson K. 2025. Colocalization of hedgehog arterivirus 1 (HhAV-1) and histologic lesions in the European hedgehog (Erinaceus europaeus) with neurological disease. Vet Pathol 62:510–521. doi:10.1177/03009858241300553 39665420

[36] Raj VS, Smits SL, Pas SD, Provacia LBV, Moorman-Roest H, Osterhaus ADME, Haagmans BL. 2012. Novel hepatitis E virus in ferrets, the Netherlands. Emerg Infect Dis 18:1369–1370. doi:10.3201/eid1808.111659 22840220 PMC3414025

[37] King CM. 2017. The chronology of a sad historical misjudgement: the introductions of rabbits and ferrets in nineteenth-century New Zealand. IREH 3:139–173. doi:10.22459/IREH.03.01.2017.09

[38] King CM. 2017. Liberation and spread of stoats (Mustela erminea) and weasels (M. nivalis) in New Zealand, 1883–1920. N Z J Ecol 41:163–177. doi:10.20417/nzjecol.41.29

[39] King C. 2023. Abundance and dynamics of small mammals in New Zealand: sequential invasions into an island ecosystem like no other. Life (Basel) 13:156. doi:10.3390/life13010156 36676105 PMC9864110

[40] Springer A, Schütte K, Brandes F, Reuschel M, Fehr M, Dobler G, Margos G, Fingerle V, Sprong H, Strube C. 2024. Potential drivers of vector-borne pathogens in urban environments: European hedgehogs (Erinaceus europaeus) in the spotlight. One Health 18:100764. doi:10.1016/j.onehlt.2024.100764 38855195 PMC11157281

[41] Martella V, Bányai K, Matthijnssens J, Buonavoglia C, Ciarlet M. 2010. Zoonotic aspects of rotaviruses. Vet Microbiol 140:246–255. doi:10.1016/j.vetmic.2009.08.028 19781872

[42] Choudhary P, Minakshi P, Ranjan K, Basanti B. 2017. Zooanthroponotic transmission of rotavirus in Haryana State of Northern India. Acta Virol 61:77–85. doi:10.4149/av_2017_01_77 28161962

[43] Zalewski A, Kołodziej-Sobocińska M, Virtanen JME, Zalewska H, Sironen T, Zub K, Nieoczym M, Popiołek M, Wereszczuk A. 2025. Drivers shaping spillover of aleutian mink disease virus introduced with American mink among native mustelids. Transbound Emerg Dis 2025:3184679. doi:10.1155/tbed/3184679 40302756 PMC12017014

[44] Knuuttila A, Aaltonen K, Virtala A-MK, Henttonen H, Isomursu M, Leimann A, Maran T, Saarma U, Timonen P, Vapalahti O, Sironen T. 2015. Aleutian mink disease virus in free-ranging mustelids in Finland - a cross-sectional epidemiological and phylogenetic study. J Gen Virol 96:1423–1435. doi:10.1099/vir.0.000081 25667324

[45] Garvey PM, Glen AS, Clout MN, Nichols M, Pech RP. 2022. Niche partitioning in a guild of invasive mammalian predators. Ecol Appl 32:e2566. doi:10.1002/eap.2566 35138656 PMC9285952

[46] Murphy EC, Dowding JE. 1994. Range and diet of stoats (Mustela erminea) in a New Zealand beech forest. N Z J Ecol:11–18.

[47] French RK, Anderson S, Cain K, Digby A, Greene TC, Miskelly CM, Muller CG, Taylor MW, Recovery Team K, Geoghegan JL, Holmes EC. 2025. Diversity and cross-species transmission of viruses in a remote island ecosystem: implications for wildlife conservation. Virus Evol 11:veae113. doi:10.1093/ve/veae113 39802822 PMC11711479

[48] Parrish CR, Holmes EC, Morens DM, Park E-C, Burke DS, Calisher CH, Laughlin CA, Saif LJ, Daszak P. 2008. Cross-species virus transmission and the emergence of new epidemic diseases. Microbiol Mol Biol Rev 72:457–470. doi:10.1128/MMBR.00004-08 18772285 PMC2546865

[49] Tompkins D M. 2018. The research strategy for a ‘predator free’ New Zealand. Proceedings of the Vertebrate Pest Conference.

[50] Dastjerdi A, Davies H, Inglese N, Holland S, Samborskiy DV, Gorbalenya AE. 2025. Intraspecific variation of the hedgehog arteriviruses, which may constitute a new genus in the subfamily heroarterivirinae of the family arteriviridae. Arch Virol 170:49. doi:10.1007/s00705-025-06231-7 39921690 PMC11807078

[51] Barratt BIP, Meenken ED, Withers TM. 2023. Characterising uncertainty in risk assessments for biological control: using case studies from New Zealand to inform future research. Biocontrol (Dordrecht) 68:101–115. doi:10.1007/s10526-023-10181-x

[52] Bolger AM, Lohse M, Usadel B. 2014. Trimmomatic: a flexible trimmer for Illumina sequence data. Bioinformatics 30:2114–2120. doi:10.1093/bioinformatics/btu170 24695404 PMC4103590

[53] Li D, Liu C-M, Luo R, Sadakane K, Lam T-W. 2015. MEGAHIT: an ultra-fast single-node solution for large and complex metagenomics assembly via succinct de Bruijn graph. Bioinformatics 31:1674–1676. doi:10.1093/bioinformatics/btv033 25609793

[54] Camacho C, Coulouris G, Avagyan V, Ma N, Papadopoulos J, Bealer K, Madden TL. 2009. BLAST+: architecture and applications. BMC Bioinformatics 10:1–9. doi:10.1186/1471-2105-10-421 20003500 PMC2803857

[55] Buchfink B, Xie C, Huson DH. 2015. Fast and sensitive protein alignment using DIAMOND. Nat Methods 12:59–60. doi:10.1038/nmeth.3176 25402007

[56] Langmead B, Salzberg S. 2013. Bowtie2. Nat Methods 9:357–359. doi:10.1038/nmeth.1923 PMC3322381 22388286

[57] Li H, Handsaker B, Wysoker A, Fennell T, Ruan J, Homer N, Marth G, Abecasis G, Durbin R, Subgroup GPDP. 2009. The sequence Alignment/Map format and SAMtools. bioinformatics 25:2078–2079. doi:10.1093/bioinformatics/btp352 19505943 PMC2723002

[58] Katoh K, Standley DM. 2013. MAFFT multiple sequence alignment software version 7: improvements in performance and usability. Mol Biol Evol 30:772–780. doi:10.1093/molbev/mst010 23329690 PMC3603318

[59] Capella-Gutiérrez S, Silla-Martínez JM, Gabaldón T. 2009. trimAl: a tool for automated alignment trimming in large-scale phylogenetic analyses. Bioinformatics 25:1972–1973. doi:10.1093/bioinformatics/btp348 19505945 PMC2712344

[60] Kalyaanamoorthy S, Minh BQ, Wong TKF, von Haeseler A, Jermiin LS. 2017. ModelFinder: fast model selection for accurate phylogenetic estimates. Nat Methods 14:587–589. doi:10.1038/nmeth.4285 28481363 PMC5453245

[61] Nguyen L-T, Schmidt HA, von Haeseler A, Minh BQ. 2015. IQ-TREE: a fast and effective stochastic algorithm for estimating maximum-likelihood phylogenies. Mol Biol Evol 32:268–274. doi:10.1093/molbev/msu300 25371430 PMC4271533

[62] Paradis E, Claude J, Strimmer K. 2004. APE: analyses of phylogenetics and evolution in R language. Bioinformatics 20:289–290. doi:10.1093/bioinformatics/btg412 14734327

[63] Yu G, Smith DK, Zhu H, Guan Y, Lam TTY. 2017. Ggtree: an R package for visualization and annotation of phylogenetic trees with their covariates and other associated data. Methods Ecol Evol 8:28–36. doi:10.1111/2041-210X.12628

[64] R Core Team R. 2013. R: A language and environment for statistical computing

[65] Wang J, Chitsaz F, Derbyshire MK, Gonzales NR, Gwadz M, Lu S, Marchler GH, Song JS, Thanki N, Yamashita RA, Yang M, Zhang D, Zheng C, Lanczycki CJ, Marchler-Bauer A. 2023. The conserved domain database in 2023. Nucleic Acids Res 51:D384–D388. doi:10.1093/nar/gkac1096 36477806 PMC9825596

