# Supplementary material for: Viral surveillance of invasive mammals in New Zealand reveals unique viral lineages reflecting their introduction history
Source: J Virol. 2025 Dec 19;100(1):e01440-25. doi: 10.1128/jvi.01440-25 (PMC12817959; doi:10.1128/jvi.01440-25)
Supplement: Fig. S1 — Maximum likelihood phylogenetic tree of the Adenoviridae, Paramyxoviridae, Picornaviridae, and Rotavirus. [file jvi.01440-25-s0001.pdf]

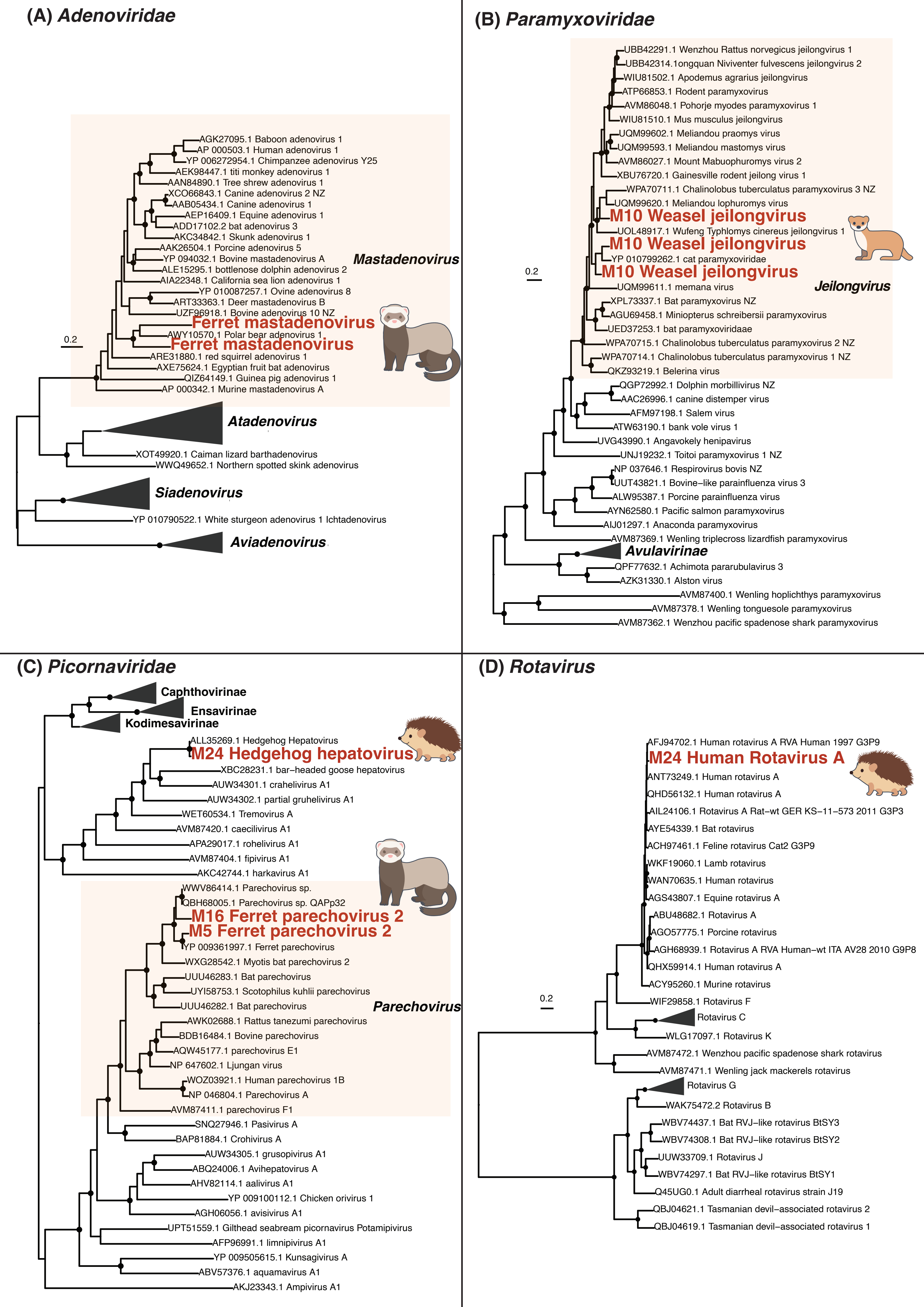

(A) Adenoviridae
AGK27095.1 Baboon adenovirus 1
AP 000503.1 Human adenovirus 1
YP 006272954.1 Chimpanzee adenovirus Y25
AEK98447.1 titi monkey adenovirus 1
AAN84890.1 Tree shrew adenovirus 1
XCO66843.1 Canine adenovirus 2 NZ
AAB05434.1 Canine adenovirus 1
AEP16409.1 Equine adenovirus 1
ADD17102.2 bat adenovirus 3
AKC34842.1 Skunk adenovirus 1
AAK26504.1 Porcine adenovirus 5
YP 094032.1 Bovine mastadenovirus A
ALE15295.1 bottlenose dolphin adenovirus 2
AIA22348.1 California sea lion adenovirus 1
YP 010087257.1 Ovine adenovirus 8
ART33363.1 Deer mastadenovirus B
UZF96918.1 Bovine adenovirus 10 NZ
Ferret mastadenovirus
AWY10570.1 Polar bear adenovirus 1
Ferret mastadenovirus
ARE31880.1 red squirrel adenovirus 1
AXE75624.1 Egyptian fruit bat adenovirus
QIZ64149.1 Guinea pig adenovirus 1
AP 000342.1 Murine mastadenovirus A
Mastadenovirus
0.2
Atadenovirus
XOT49920.1 Caiman lizard barthadenovirus
WWQ49652.1 Northern spotted skink adenovirus
Siadenovirus
YP 010790522.1 White sturgeon adenovirus 1 Ichtadenovirus
Aviadenovirus
(B) Paramyxoviridae
UBB42291.1 Wenzhou Rattus norvegicus jeilongvirus 1
UBB42314.1ongquan Niviventer fulvescens jeilongvirus 2
WIU81502.1 Apodemus agrarius jeilongvirus
ATP66853.1 Rodent paramyxovirus
AVM86048.1 Pohorje myodes paramyxovirus 1
WIU81510.1 Mus musculus jeilongvirus
UQM99602.1 Meliandou praomys virus
UQM99593.1 Meliandou mastomys virus
AVM86027.1 Mount Mabuophuromys virus 2
XBU76720.1 Gainesville rodent jeilong virus 1
WPA70711.1 Chalinolobus tuberculatus paramyxovirus 3 NZ
UQM99620.1 Meliandou lophuromys virus
M10 Weasel jeilongvirus
UOL48917.1 Wufeng Typhlomys cinereus jeilongvirus 1
M10 Weasel jeilongvirus
YP 010799262.1 cat paramyxoviridae
M10 Weasel jeilongvirus
UQM99611.1 memana virus
Jeilongvirus
0.2
XPL73337.1 Bat paramyxovirus NZ
AGU69458.1 Miniopterus schreibersii paramyxovirus
UED37253.1 bat paramyxoviridaae
WPA70715.1 Chalinolobus tuberculatus paramyxovirus 2 NZ
WPA70714.1 Chalinolobus tuberculatus paramyxovirus 1 NZ
QKZ93219.1 Belerina virus
QGP72992.1 Dolphin morbillivirus NZ
AAC26996.1 canine distemper virus
AFM97198.1 Salem virus
ATW63190.1 bank vole virus 1
UVG43990.1 Angavokely henipavirus
UNJ19232.1 Toitoi paramyxovirus 1 NZ
NP 037646.1 Respirovirus bovis NZ
UUT43821.1 Bovine−like parainfluenza virus 3
ALW95387.1 Porcine parainfluenza virus
AYN62580.1 Pacific salmon paramyxovirus
AIJ01297.1 Anaconda paramyxovirus
AVM87369.1 Wenling triplecross lizardfish paramyxovirus
Avulavirinae
QPF77632.1 Achimota pararubulavirus 3
AZK31330.1 Alston virus
AVM87400.1 Wenling hoplichthys paramyxovirus
AVM87378.1 Wenling tonguesole paramyxovirus
AVM87362.1 Wenzhou pacific spadenose shark paramyxovirus
(C) Picornaviridae
Caphthovirinae
Ensavirinae
Kodimesavirinae
ALL35269.1 Hedgehog Hepatovirus
M24 Hedgehog hepatovirus
XBC28231.1 bar−headed goose hepatovirus
AUW34301.1 crahelivirus A1
AUW34302.1 partial gruhelivirus A1
WET60534.1 Tremovirus A
AVM87420.1 caecilivirus A1
APA29017.1 rohelivirus A1
AVM87404.1 fipivirus A1
AKC42744.1 harkavirus A1
WWV86414.1 Parechovirus sp.
QBH68005.1 Parechovirus sp. QAPp32
M16 Ferret parechovirus 2
M5 Ferret parechovirus 2
YP 009361997.1 Ferret parechovirus
WXG28542.1 Myotis bat parechovirus 2
UUU46283.1 Bat parechovirus
UYI58753.1 Scotophilus kuhlii parechovirus
UUU46282.1 Bat parechovirus
Parechovirus
AWK02688.1 Rattus tanezumi parechovirus
BDB16484.1 Bovine parechovirus
AQW45177.1 parechovirus E1
NP 647602.1 Ljungan virus
WOZ03921.1 Human parechovirus 1B
NP 046804.1 Parechovirus A
AVM87411.1 parechovirus F1
SNQ27946.1 Pasivirus A
BAP81884.1 Crohivirus A
AUW34305.1 grusopivirus A1
ABQ24006.1 Avihepatovirus A
AHV82114.1 aalivirus A1
YP 009100112.1 Chicken orivirus 1
AGH06056.1 avisivirus A1
UPT51559.1 Gilthead seabream picornavirus Potamipivirus
AFP96991.1 limnipivirus A1
YP 009505615.1 Kunsagivirus A
ABV57376.1 aquamavirus A1
AKJ23343.1 Ampivirus A1
(D) Rotavirus
AFJ94702.1 Human rotavirus A RVA Human 1997 G3P9
M24 Human Rotavirus A
ANT73249.1 Human rotavirus A
QHD56132.1 Human rotavirus A
AIL24106.1 Rotavirus A Rat−wt GER KS−11−573 2011 G3P3
AYE54339.1 Bat rotavirus
ACH97461.1 Feline rotavirus Cat2 G3P9
WKF19060.1 Lamb rotavirus
WAN70635.1 Human rotavirus
AGS43807.1 Equine rotavirus A
ABU48682.1 Rotavirus A
AGO57775.1 Porcine rotavirus
AGH68939.1 Rotavirus A RVA Human−wt ITA AV28 2010 G9P8
QHX59914.1 Human rotavirus A
ACY95260.1 Murine rotavirus
0.2
WIF29858.1 Rotavirus F
Rotavirus C
WLG17097.1 Rotavirus K
AVM87472.1 Wenzhou pacific spadenose shark rotavirus
AVM87471.1 Wenling jack mackerels rotavirus
Rotavirus G
WAK75472.2 Rotavirus B
WBV74437.1 Bat RVJ−like rotavirus BtSY3
WBV74308.1 Bat RVJ−like rotavirus BtSY2
UUW33709.1 Rotavirus J
WBV74297.1 Bat RVJ−like rotavirus BtSY1
Q45UG0.1 Adult diarrheal rotavirus strain J19
QBJ04621.1 Tasmanian devil−associated rotavirus 2
QBJ04619.1 Tasmanian devil−associated rotavirus 1

Supplementary Figure S1. Maximum likelihood phylogenetic tree of the (A) *Adenoviridae*, (B) *Paramyxoviridae*, (C) *Picornaviridae* and (D) *Rotavirus*. Viruses identified in this study are shown in red and related viruses are shown in black. Black circles on nodes indicate bootstrap support values of >90%. Branches are scaled according to the number of amino acid substitutions per site, shown in the scale bar. The trees are midpoint rooted for purposes of clarity only.
