# Supplementary material for: Viral surveillance of invasive mammals in New Zealand reveals unique viral lineages reflecting their introduction history
Source: J Virol. 2025 Dec 19;100(1):e01440-25. doi: 10.1128/jvi.01440-25 (PMC12817959; doi:10.1128/jvi.01440-25)
Supplement: Fig. S2 — Annotated genome structures. [file jvi.01440-25-s0002.pdf]

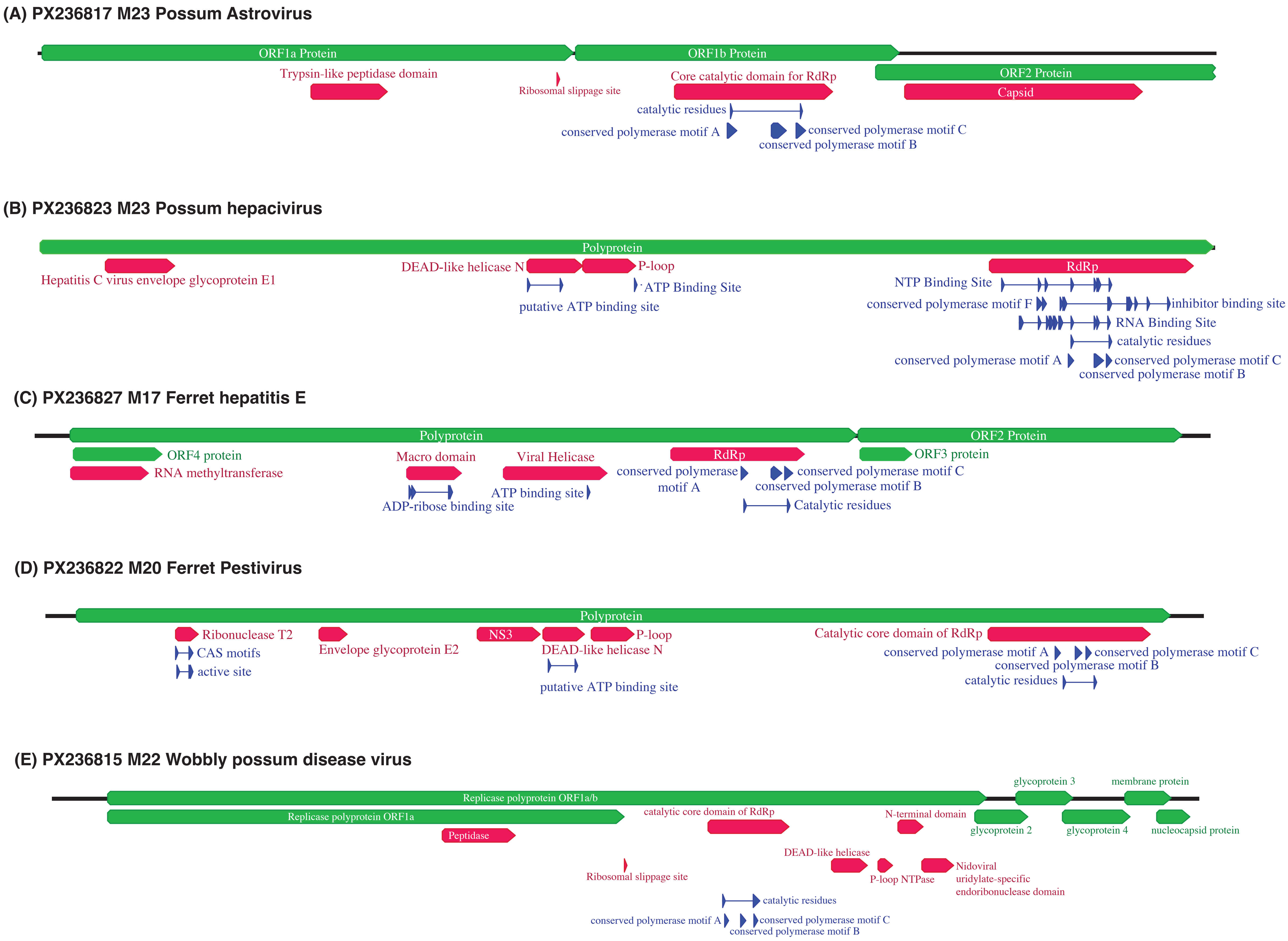

Supplementary Figure S2. Annotated genome structures of five viruses found in this study, based on BLASTx, BLASTp and conserved domain searches (CD-search).
